# Predictors for replanning in loco-regionally advanced nasopharyngeal carcinoma patients undergoing intensity-modulated radiation therapy: a prospective observational study

**DOI:** 10.1186/1471-2407-13-548

**Published:** 2013-11-16

**Authors:** DanFang Yan, SenXiang Yan, QiDong Wang, XinBiao Liao, ZhongJie Lu, YiXiang Wang

**Affiliations:** 1 Department of Radiation Oncology, the First Affiliated Hospital, College of Medicine, Zhejiang University, 79 Qingchun Road, Hangzhou, Zhejiang 310003, PR China; 2 Department of Radiology, the First Affiliated Hospital, College of Medicine, Zhejiang University, Hangzhou, Zhejiang 310003, China; 3 Department of Imaging and Interventional Radiology, The Chinese University of Hong Kong, Prince of Wales Hospital, Shatin, Hong Kong, China

**Keywords:** Nasopharyngeal carcinoma, Intensity-modulated radiotherapy, Replanning, Body mass index, Apparent diffusion coefficients

## Abstract

**Background:**

Replanning in intensity-modulated radiotherapy (IMRT) has been reported to improve quality of life and loco-regional control in patients with nasopharyngeal cancer (NPC). Determination of the criteria for replanning is, however, urgently needed. We conducted a prospective study to determine when and for what type of patients is replanning preferred through weekly repeat computed tomography (CT) imaging during the course of IMRT.

**Methods:**

We recruited 20 patients who were diagnosed as having loco-regionally advanced, non-metastatic stage III or IVa NPC and treated with concurrent platinum-based chemoradiotherapy (CRT) using IMRT. Patients received CT simulation (sim-CT) and plain magnetic resonance imaging (MRI) plus diffusion-weighted imaging (DWI) weekly for five consecutive weeks. The gross tumor volume (GTV) and clinical target volume (CTV) were delineated and recorded weekly based on the CT-CT fusion. The relationship between GTV/CTV reduction and clinical characteristics of the patients were assessed using Pearson correlation test.

**Results:**

GTV and CTV decreased during the treatment by 36.03 mL (range, 10.91–98.82 mL) and 76.79 mL (range, 33.94–125.14 mL), respectively, after 25 fractions of treatment. The percentage reductions from their initial volume were 38.4% (range, 25.3–50.7%) and 11.8% (range, 6.7–18.3%), respectively. The greatest reductions in GTV and CTV were observed at the fourth week (i.e., upon completion of 20 fractions), compared to pre-treatment sim-CT. Weight loss and CTV reduction were significantly correlated with pre-treatment body mass index (BMI ) (*r* = 0.58, *P* = 0.012, and *r* = 0.48, *P* = 0.046, respectively). However, no significant correlation was observed between CTV reduction and initial tumor volume. In addition, GTV reduction was not significantly correlated with pre-treatment tumor volume (*P* = 0.65), but negatively correlated with pre-treatment tumor apparent diffusion coefficient (ADC) values (*r* = −0.46, *P* = 0.042).

**Conclusions:**

Our results indicate that the most appropriate replanning time is after 20 fractions of treatment, and pre-treatment BMI and ADC are potential predictive factors for the determination of replanning during IMRT.

## Background

Nasopharyngeal carcinoma (NPC) is a relatively common head and neck malignancy in southern China. The annual crude incidence rate of NPC was reported to be 13.4 per 100,000 in Hong Kong, and the World Health Organization (WHO) estimated 80,043 new cases per year worldwide [1]. Numerous studies have demonstrated that intensity-modulated radiation therapy (IMRT) can reduce radiation toxicity and achieve better local control in patients with NPC [2,3]. However, the optimized IMRT plan frequently has a significant dose gradient change around the target margin. Therefore, accuracy in the target range is essential and is a heightened concern in the treatment plan.

The majority of NPC patients are at the locally advanced stage at first diagnosis. Concurrent platinum-based chemotherapy with IMRT is the standard treatment for locally advanced NPC. During the course of IMRT, especially with concurrent chemoradiotherapy (CRT), NPC has been considered to be not only radiosensitive but also chemosensitive [4,5]. Most NPC patients will experience changes in anatomic structures due to obvious tumor shrinkage and/or weight loss [6], which leads to varying degrees of deviation in dose distributions to both tumors and organs at risk (OARs). Recently, the importance of replanning during IMRT has received more attention. Hansen *et al.* found that the hybrid IMRT plans (without replanning) demonstrated reduced doses to target volumes and increased doses to critical structures, compared to IMRT with replanning [7]. In hybrid IMRT plans, doses up to 95% of the planning target volumes (PTVs) for the GTV and clinical target volume (CTV) were reduced in 92% of patients, by 0.8–6.3 Gy and 0.2–7.4 Gy, respectively. The maximum dose to the spinal cord increased in all patients by 0.2–15.4 Gy, and the maximum dose to brainstem increased by 0.6–8.1 Gy in 85% of patients in hybrid IMRT plans. Similar results were also reported by Height *et al.*[8]. In addition, Zhao *et al*. [9] studied the benefits to clinical outcomes from replanning and demonstrated that replanning could not only alleviate the late effects, but also improve 3-year local progression-free survival.

It has been proved that replanning during IMRT is essential, especially for patients receiving CRT. However, repeat CT scan and replanning are time-consuming for both physicians and oncologists, and not all institutes have proper conditions to replan for every patient. Therefore, it is important to determine when and for what types of patients are repeated CT and replanning preferred during IMRT. To address these questions, we assessed the dynamic changes in GTV and CTV on weekly repeat CT images during the course of IMRT for loco-regionally advanced NPC patients and investigated whether the related factors could predict the volume reductions observed in this study.

## Methods

### Study patients

Twenty consecutive patients with loco-regionally advanced NPC were enrolled in this prospective study. The study was approved by the Ethics Committee of the First Affiliated Hospital of College of Medicine at Zhejiang University, and informed consent was obtained from all participants. All 20 patients underwent an endoscopic examination for a clinically suspected lesion in the nasopharynx and pathology was obtained from the primary tumor before CRT. Subsequent staging work-up revealed no evidence of distant metastasis in all patients.

### Image acquisition

Before treatment, all patients underwent head-and-neck immobilization with thermoplastic masks (MEDTEC, Orange City, IA) and CT simulation. All CT scans were obtained on a Siemens Sensation Open scanner (Erlangen, Germany) using 3-mm slice thickness with contrast enhancement. Contrast-enhanced T1- and plain T2-weighted MRI plus diffusion-weighted imaging (DWI) were acquired before treatment. Thereafter, all patients received sim-CT and plain MRI plus DWI weekly for five consecutive weeks (i.e., at the completion of 5, 10, 15, 20, and 25 fractions).

### Definition of target volumes

Based on the optimized CT images, one principal oncologist was responsible for delineating the target volume for the studied NPC patients. The GTV included the primary lesion and cervical lymphadenopathy shown on CT and MRI. The CTV was defined as the GTV plus a margin of potential microscopic spread, which included the nasopharynx, retropharyngeal nodes, clivus, skull base, pterygoid fossae, parapharyngeal space, inferior sphenoid sinus, posterior third of the nasal cavity and maxillary sinuses, and cervical lymphatics. The pre-treatment GTV and CTV were labeled GTV_0_ and CTV_0_, respectively, and the following GTVs and CTVs obtained weekly for five weeks were labeled GTV_1-5_ and CTV_1-5_ accordingly. After weekly repeat CT scan, CT-CT image fusions were performed between pre-treatment CT (CT_0_) and repeat CT images (CT_1_, CT_2_, CT_3_, CT_4_, and CT_5_). The repeat CT images were aligned to the initial treatment planning CT using rigid bony co-registration, while accepting minor registration mismatch due to inadvertent distortional set up discrepancy. The delineation of GTV_0_ and CTV_0_ were then copied onto the repeat CT images. Subsequently, the oncologist who was responsible for delineating GTV_0_ and CTV_0_ further modified and recorded the following weekly GTV and CTV according to anatomical changes and mass shrinkage.

### Weight and tumor ADC acquisition

The height and weight of each patient were obtained and the BMI was calculated on the day of the initial planning CT scan. The patients’ weight throughout CRT was recorded weekly on the same days as CT re-scanning.

The MRI studies were performed on a Philips 3.0 T Intera Master combining a standard head coil, two-channel dedicated surface neck coil and spine coil. DWI was performed using the multiple-section spin-echo single-shot echoplanar sequence in the transverse plane, using a matrix of 96 × 96, TR/TE = 2947.1 ms/43.3 ms, b-values of 0 and 1,500 s/mm2, FOV of 260 mm × 260 mm, NSA of 6 times. The DWI was analyzed on a workstation (Agfa-Gevaert, Mortsel, Belgium) by an experienced radiologist who was blind to this study. The regions of interest (ROIs) were placed over the entire lesions except for obvious necrotic or cystic components on DWI. Subsequently, apparent diffusion coefficient (ADC) values for all ROIs were obtained directly from an ADC map, which was reconstructed using b values of 0 and 1,500 s/mm^2^. Manually circumscribed ROIs were used to quantitate the primary tumor on each slice, and an average value for these ROIs was calculated for use as a lesion’s final ADC value.

### Treatment planning and delivery

All patients received IMRT with simultaneous integrated boost (SIB-IMRT). A second IMRT plan was generated from the optimized CT_4_ (scan at the completion of 20 fractions) to complete the planned course of treatment though we performed weekly repeat CT scan. SIB-IMRT was delivered to a total dose of 6540–7412 cGy/30-34 F for planning GTV (PGTV) and 5264 cGy/28 F-6016 cGy/32 F for PTV. All 20 patients received cisplatin 80 mg/m^2^ i.v. on days 1, 22, and 43 during IMRT.

### Statistical analysis

Statistical analysis was performed using SAS 9.0 software package. The differences in weight loss between BMI ≥25 and <25 groups were calculated by independent samples *t* test. Comparisons were made by paired *t* test between the volumes on the scan at a particular time point and the scan performed on the preceding week (i.e., pretreatment [week 0] with week 1, week 2 with week 3, and so on). Pearson correlation was performed to determine whether CTV reduction was related to pre-treatment BMI and initial tumor mass, and GTV shrinkage to pre-treatment tumor ADC and initial tumor mass. A *P*-value less than 0.05 was considered statistically significant.

## Results

### Patient characteristics

The 20 enrolled patients included 13 men and 7 women with a mean age of 49 years (range, 37–61 years). Eighteen patients presented with stage III disease and 2 patients with IVa disease according to the 7^th^ Edition of the American Joint Committee on Cancer (AJCC). Histologically, 11 patients had well differentiated non-keratinizing, 4 had poorly differentiated non-keratinizing, 4 had poorly differentiated squamous cell, and 1 had unclassified carcinomas. Of the 20 patients, the initial median weight and BMI was 60 kg (range, 50–86 kg) and 23.5 (range, 19–26.5), respectively. Seven of 20 patients were overweight (BMI ≥ 25), and the other 13 were normal weight (18.5 ≤ BMI < 25).

### Weight loss of the patients

Most patients experienced significant weight loss during the course of CRT. Compared with the baseline, mean weight loss at treatment completion of 25 fractions was 7 kg, which corresponded to a percentage of 13.6% (range, 3.9–25.5%). Over the entire treatment course, the greatest weight loss occurred at the end of the second week, which corresponded to the completion of 10 fractions of radiation. Moreover, weight loss in patients with BMI ≥ 25 was significantly greater than that in patients with BMI < 25 (*t* = 3.39, *P* = 0.0033), and a significant correlation was observed between weight loss and pre-treatment BMI (*r* = 0.58, *P* = 0.012), indicating that patients with higher BMI experience greater weight loss during CRT.

### CTV changes

**Figure 1:**
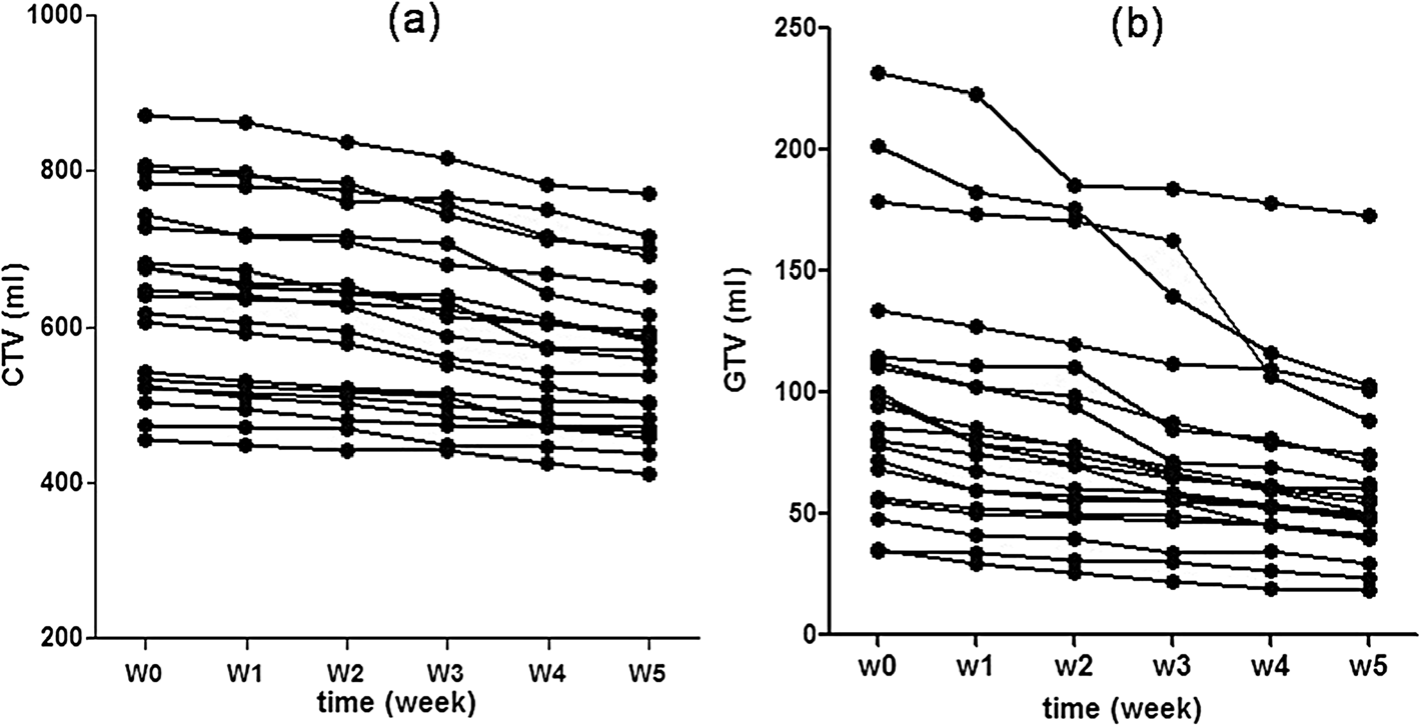
**Changes in CTV (a) and GTV (b) each week in 20 nasopharyngeal cancer patients during the course of CRT.***CTV: clinical target volume; GTV: gross target volume; CRT: chemoradiotherapy.*

**Figure 2:**
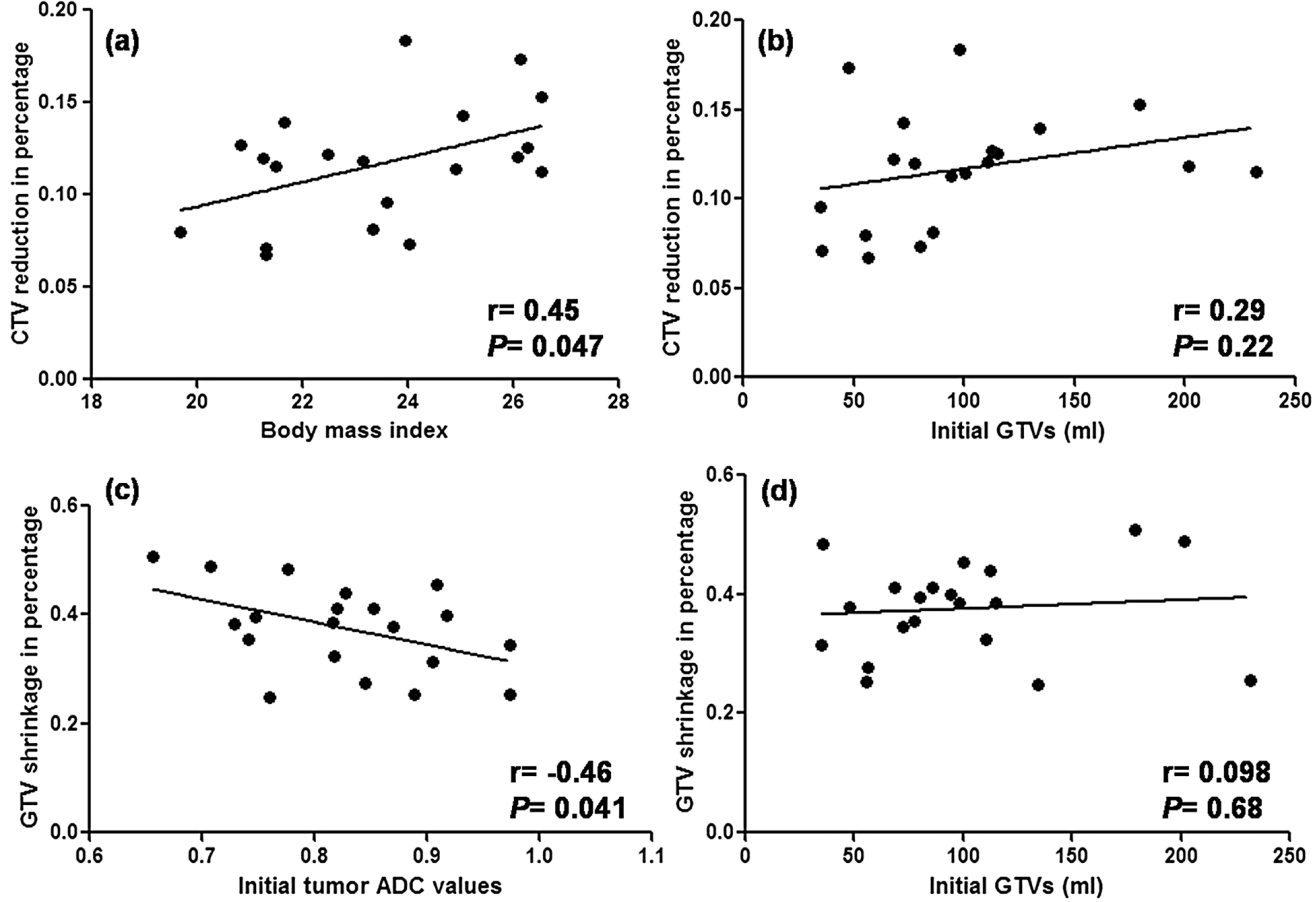
**Pearson correlation between CTV reduction and BMI or initial tumor mass, between GTV and ADC or initial tumor mass. (a)**: Correlation between BMI and CTV reduction; **(b)**: correlation between initial tumor mass and CTV reduction; **(c)**: correlation between pre-treatment tumor ADC and GTV reduction; **(d)**: correlation between initial tumor mass and GTV shrinkage. CTV: clinical target volume; GTV: gross target volume; BMI: body mass index; ADC: apparent diffusion coefficient.

**Figure 3:**
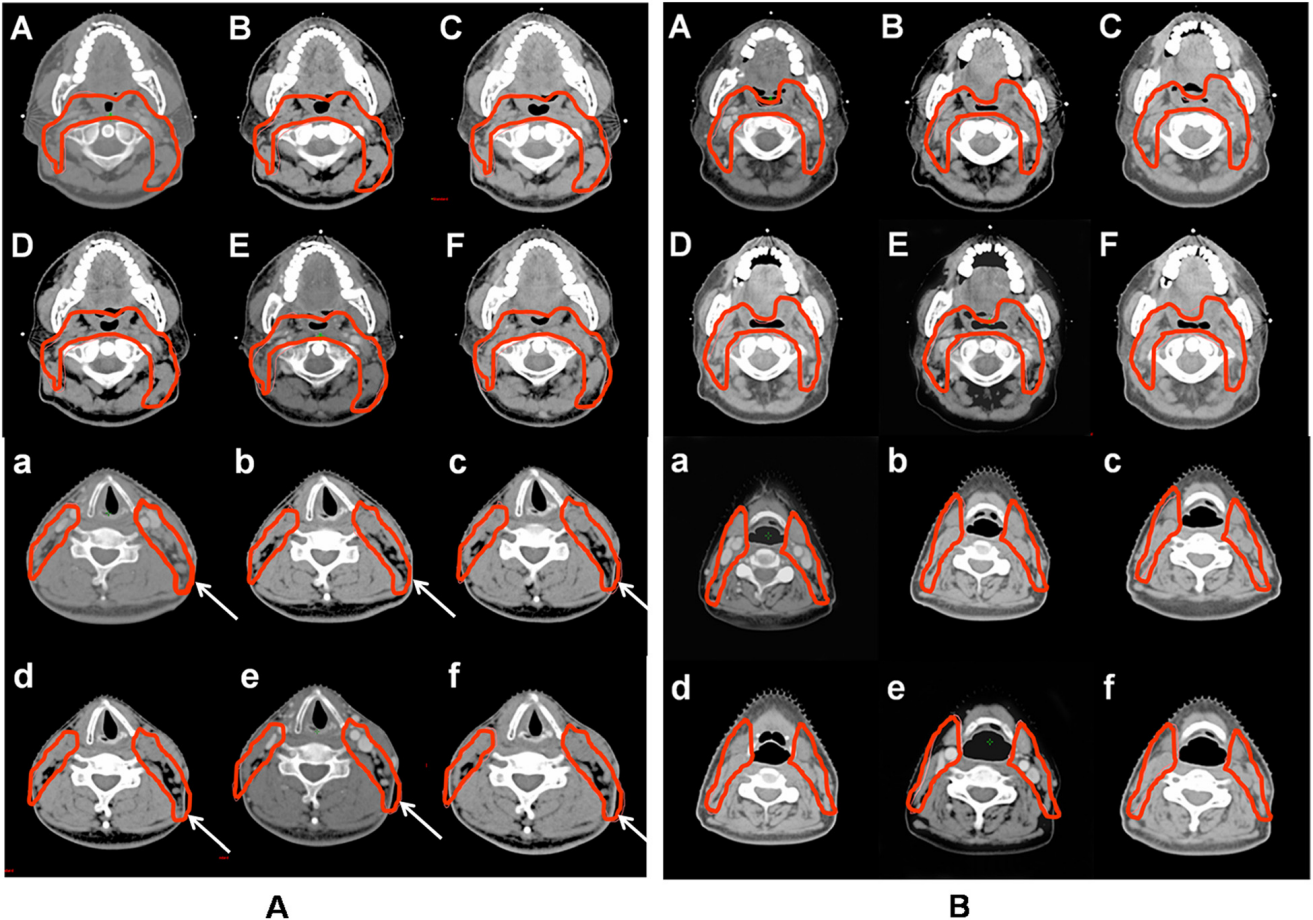
**Comparison of the anatomical structure changes in an overweight patient and a normal BMI patient. (A)**: a patient with BMI = 26.5; **(B)**: a patient with BMI = 19.7. The arrows show the obvious anatomical structure changes during CRT on cervical slices. The upper and the lower panels show representive oropharyngeal and cervical slices at different time points from week 0 to week 5, respectively. *BMI: body mass index; CTV: clinical target volume; CRT: chemoradiotherapy.*

Compared with the baseline, mean CTV reduction after 25 fractions was 76.79 mL, corresponding to a percentage of 11.9% (range, 6.7–22.9%). Comparing the volume change of each week with that of the preceding week showed that the greatest CTV reduction of 23.63 mL (3.7%) occurred from week 3 to week 4 (Table 1 and Figure 1a). In addition, CTV reduction was significantly correlated with pre-treatment BMI (*r* = 0.45, *P* = 0.047) (Figure 2a), indicating that patients with higher BMI had a greater CTV reduction. We also found a positive correlation between weight loss and CTV reduction during CRT (*r* = 0.71, *P* = 0.0009). However, no significant correlation was observed between CTV reduction and initial GTV (GTV0) (Figure 2b). We compared the anatomical structure changes on oropharyngeal and cervical slices between a patient with a BMI of 26.5 (Figure 3A) and a patient with a BMI of 19.7 (Figure 3B) when the original CTV was contoured on oropharyngeal and cervical slices. The anatomical structure changes, especially on cervical slices, were much greater in the overweight patient than in the normal weight patient.

**Table 1:** Comparisons of the absolute and percentage reductions in weekly CTV and GTV

**Target volume**	**Comparison**	**Absolute reduction (mean + SD, mL)**	**Percentage reduction (mean, %)**	***t***	***P***
CTV	CTV_0_* vs CTV_1_	10.95 ± 1.35	1.8	8.07	<0.0001
	CTV_1_ vs CTV_2_	10.91 ± 2.18	1.8	2.18	<0.0001
	CTV_2_ vs CTV_3_	12.27 ± 3.17	2.9	5.44	<0.0001
	CTV_3_ vs CTV_4_	23.63 ± 4.03	3.7	5.86	<0.0001
	CTV_4_ vs CTV_5_	13.69 ± 2.12	2.2	6.46	<0.0001
GTV	GTV_0_* vs GTV_1_	8.80 ± 1.25	10.3	7.05	<0.0001
	GTV_1_ vs GTV_2_	5.98 ± 1.74	6.8	3.44	0.0027
	GTV_2_ vs GTV_3_	8.79 ± 2.14	9.9	4.10	0.0006
	GTV_3_ vs GTV_4_	8.01 ± 2.75	10.5	2.91	0.009
	GTV_4_ vs GTV_5_	6.20 ± 0.93	9.1	6.63	<0.0001

*The pretreatment volumes were labeled week 0.

### GTV changes

**Figure 4:**
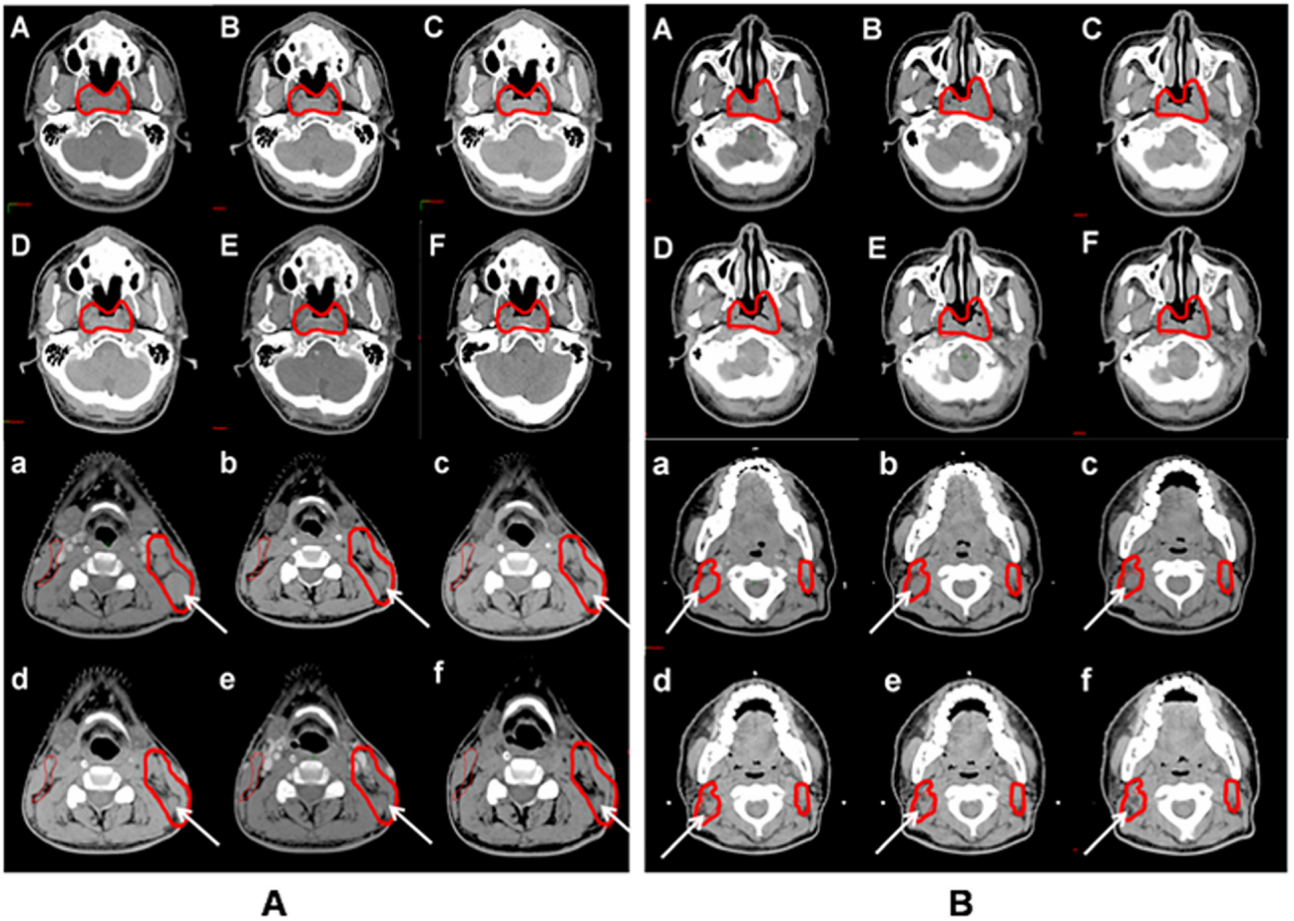
**Comparison of GTV shrinkage in two patients with different tumor ADC. (A)**: a patient with a tumor ADC of 0.7 × 10-3 mm/s; **(B)** a patient with an ADC of 0.89 × 10-3 mm/s. The arrows indicate cervical lymph node shrinkage in the patients during CRT. The upper and the lower panels show representive primary tumor and cervical lymph node slices at different time points from week 0 to week 5, respectively. *GTV: gross target volume; ADC: apparent diffusion coefficient; CRT: chemoradiotherapy.*

Compared with pre-treatment, mean GTV shrinkage after 25 fractions was 36.03 mL, corresponding to a percentage of 37.6% (range, 25.3–50.7%). The weekly absolute and percentage reductions in GTV are listed in Table 1. The two greatest percentage reductions in GTV were 10.3% at week 1 and 10.5% at week 4 (Table 1 and Figure 1b). GTV shrinkage was significantly correlated with the pre-treatment ADC values (*r* = −0.46, *P* = 0.041) (Figure 2c), suggesting that greater GTV reductions occurred in those patients with lower initial ADCs. However, no significant correlation was observed between GTV reduction and pre-treatment tumor volume (*P* = 0.68) (Figure 2d). Representative GTV reductions of the primary tumor and cervical lymph nodes are shown in Figure 4. Figure 4(A) corresponds to a patient with a tumor ADC of 0.7 × 10^-3^ mm/s, and Figure 4(B) corresponds to a patient with an ADC of 0.89 × 10^-3^ mm/s. We observed that patients with lower tumor ADCs had relatively greater GTV shrinkage.

### Weekly changes in tumor ADC

**Figure 5:**
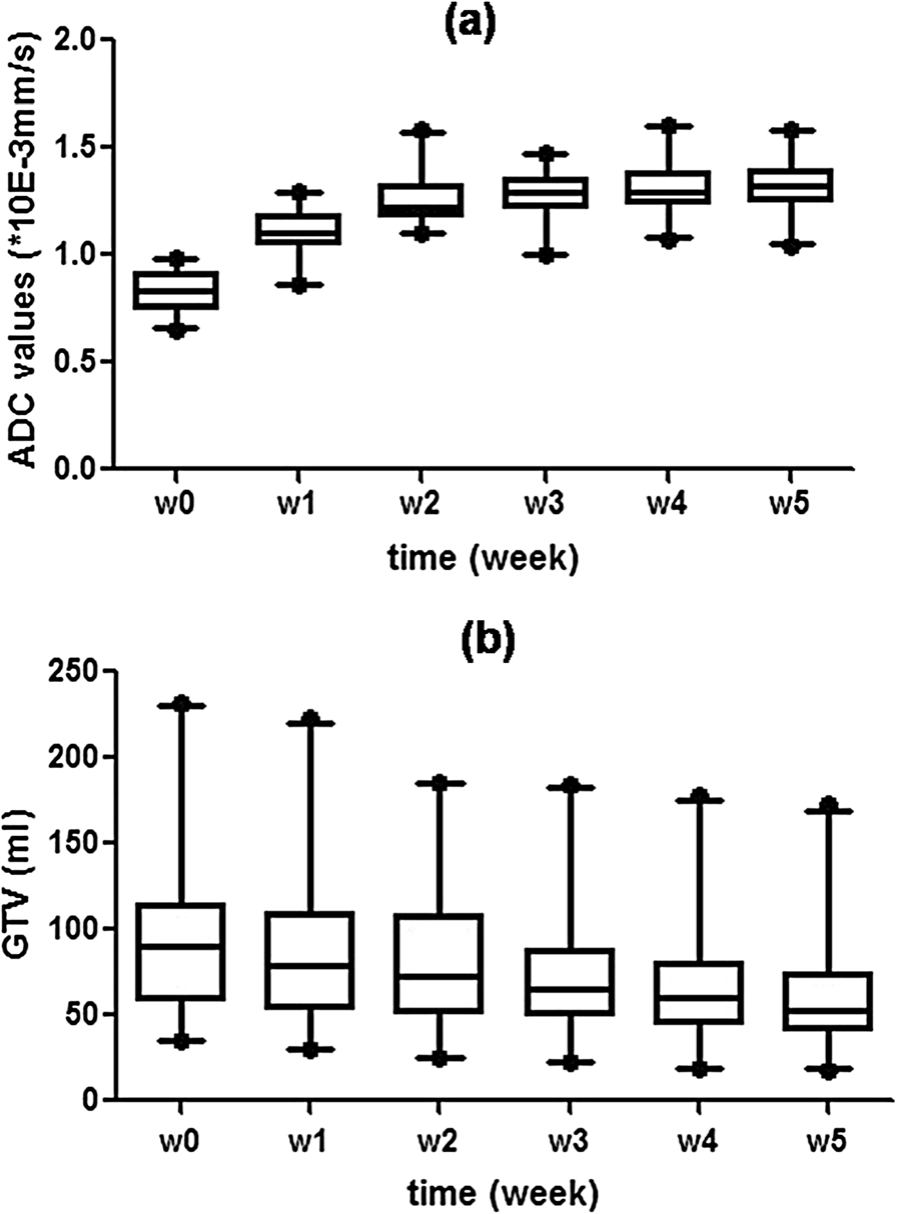
**The mean absolute changes in ADC (a) values and GTV (b).** Error bars represent 95% confidence intervals. *GTV: gross target volume; ADC: apparent diffusion coefficient.*

Mean ADC values increased significantly during the first two weeks of CRT, from 0.84 × 10^-3^ mm/s to 1.25 × 10^-3^ mm/s. Thereafter, ADCs continued to increase and reached a relatively flat plateau from weeks 3 to 5 (Figure 5 a). During the last three weeks, ADCs only increased by 0.031 × 10^-3^ mm/s. In contrast to the ADCs, the GTVs reduced steadily every week (Figure 5b).

## Discussion

In our institute, we routinely perform repeat CT and replanning for all NPC patients during IMRT. The time point for replanning is generally selected at the completion of 20 or 25 fractions. However, there are still no theoretical data to support this. Because performing repeat CT and replanning for each patient is time-consuming, criteria are urgently needed for determining which patients need replanning during IMRT and when. For the first time to the best of our knowledge, we have reported dynamic changes in GTVs and CTVs, and their correlation with potential predictive factors such as pre-treatment tumor mass, tumor ADCs, and patient BMI in loco-regionally advanced NPC patients undergoing IMRT.

Given that significant anatomical changes occurred frequently from the second to the fifth week of treatment based on our experience and other previous studies [8,10], we performed repeat CT scanning weekly for five weeks during a 32- or 33-fraction IMRT. Comparing patients with BMI ≥ 25 to patients with BMI < 25, we found that overweight patients were more likely to experience weight loss during CRT. We speculate that overweight patients have more cervical adipose tissue, and therefore the neck circumference of these patients is more easily affected by weight loss during CRT. Height *et al.* reported the median weight loss post 40–50 Gy of radiation was 3%, although all enrolled patients participated in an early nutritional intervention program to minimize weight loss via tube feeding [8]. Because none of the patients received tube feeding in our study, the weight loss observed was much greater than that in the study of Height et al. We observed a mean weight loss of 13.6% (range, 3.9–25.5%) after five weeks of radiation. This weight loss was considered to be associated with the side effects of CRT, such as oropharyngeal mucositis and gastrointestinal reactions, which were comparable among the 20 patients and thus not taken into account in the final analysis.

It has been recognized that most patients with head and neck cancers receiving radiotherapy experience changes in anatomical structures mainly due to shrinkage of primary tumors, metastatic nodal masses, and body contour caused by profound body weight loss [7,11-14]. In the present study, we assessed the relationship between CTV reduction and original GTV or pre-treatment BMI and found a positive correlation between CTV reduction and BMI. This result indicates that patients with a higher BMI are more susceptible to changes in body weight during CRT and consequently experience a greater reduction in CTV. Therefore, patients with BMI ≥ 25 should be recommended for repeat CT scanning and replanning. However, there was no obvious correlation between CTV reduction and original GTV, indicating that original tumor mass probably is not a sensitive predictor of anatomical structure changes.

Because it is obviously impractical to perform replanning weekly, it is also important to determine the appropriate time point for replanning. We performed repeat CT weekly and observed the most significant CTV reduction at the fourth week (e.g., at the completion of 20 fractions), not only in terms of absolute volume reduction but also percentage compared with the preceding week. The peak time of this reduction is consistent with the peak time of oropharyngeal mucositis caused by chemoradiotherapy.

We hypothesized that GTV shrinkage was most likely related to the size of pre-treatment tumor mass (including primary mass and metastatic lymph nodes). Interestingly, we found that GTV shrinkage was not correlated with pre-treatment GTV, but significantly correlated with pre-treatment tumor ADC. In other words, tumor ADC may be more sensitive than initial tumor mass for predicting tumor shrinkage in response to CRT. DWI is considered a functional technique because it is sensitive to the molecular motion of water, reflecting the viability and structure of tissue on a cellular level and providing information about the tissue functional structure at a microscopic level [15,16]. Many studies have demonstrated that malignant lesions have low ADCs due to the restriction of water molecules. A possible mechanism for this is that hypercellularity, which corresponds to reduced extracellular and intracellular volume in malignant lesions, inhibits water motion [17-19].

The ADC value has been used to predict and detect tumor responses to therapy [20-23]. Kim *et al.*[20] investigated 33 patients with head and neck squamous cell carcinomas who had DWI before, during, and after CRT. They found that pre-treatment ADC value and changes in ADC within the first week were significantly different between complete responders and partial responders, suggesting that ADC can be used as an early predictor of response to CRT. However, some other studies showed that ADC at mid-treatment, instead of pre-treatment, can predict the final tumor response [22,23]. However, we found a negative correlation between pre-treatment ADC and percentage reduction in GTV. One possible explanation for the above findings is that metastatic lymph nodes were diagnosed morphologically in this study as meeting the criteria of ≥1 cm in short axis or presence of necrosis. Those metastatic nodes with higher ADC values suggested existence of either macroscopic or microscopic necrosis within the lesions, which was a sign of tumor hypoxia. It is well-known that hypoxic tumor cells are more resistant to cytotoxic treatment including radiotherapy. Further study is ongoing in our institute on tumor hypoxia and treatment response using DWI and PET scan.

When replanning is performed during IMRT based on obvious anatomic changes, it is well accepted for CTV recontouring, but not for GTV recontouring. It remains controversial whether GTV should be recontoured or remain at the initial volume when primary tumors or cervical lymph nodes shrink noticeably during treatment. Some physicians choose to maintain the original GTV for two reasons: 1) they are unsure whether it is safe (in terms of local-regional control) to decrease the GTV; and 2) they have had excellent local-regional control for head and neck cancer patients treated with IMRT in their institutes [7,24]. However, Zhao and colleagues chose to recontour GTV on repeat CTs and observed excellent local-regional control and alleviation of acute/late toxicity due to a smaller volume of high-dose treatment [9,25]. In our institute, we routinely recontour GTV in replanning IMRT, because the tumor mass in NPC shrinks either in a concentric (like the metastaic cervical nodes without extracapsular extension) or a honeycomb (like the primary lesion invading the cranial base) manner, recontouring is needed in situation of concentric tumor shrinkage.

In this study, an immediate and substantial increase in ADC values was observed during the first two weeks before a relatively flat plateau was reached. Thereafter, a smooth decline was observed in mean, absolute GTVs during treatment (Figure 2). In comparisons of GTV reduction, both in absolute volumes and in percentages, at each week relative to the preceding week, significant changes were found at weeks 1, 3, and 4. This result suggests that tumor cells begin to lose viability on the third week, and GTV recontouring, if it is needed, should be performed at least after the third week, although GTV started shrinking after the first-week treatment. Considering these results in combination with the characteristics of CTV changes, week 4 should be an appropriate time for replanning during CRT.

We acknowledge that this study has several limitations. First, the small sample size limited our ability to use multivariate logistic regression models to determine other predictive factors for replanning. Second, because this study was performed at a single center and was relatively homogeneous, multicenter and large-scale studies are warranted.

## Conclusions

Our study demonstrates that patients with high pre-treatment BMI are more likely to experience CTV reduction and those with lower tumor ADCs are prone to greater GTV shrinkage. Pre-treatment BMI and tumor ADCs have potential as predictive factors for replanning. Moreover, this study also suggests that the most appropriate replanning time is after 20 fractions of treatment.

## Abbreviations

IMRT: Intensity-modulated radiotherapy; NPC: Nasopharyngeal cancer; CT: Computed tomography; CRT: Chemoradiotherapy; MRI: magnetic resonance imaging; DWI: Diffusion-weighted imaging; GTV: Gross tumor volume; CTV: Clinical target volume; PTV: Planning target volume; OARs: Organs at risk; BMI: Body mass index; ADC: Apparent diffusion coefficient; ROIs: Regions of interest; SIB-IMRT: IMRT with simultaneous integrated boost; WHO: World Health Organization; AJCC: American Joint Committee on Cancer.

## Competing interests

The authors declare that they have no competing interests. No reimbursements, fees, funding, or salary was received from an organization that may in any way gain or lose financially from the publication of this manuscript now and in the future. We don’t hold any stocks or shares in an organization that may in any way gain or lose financially from the publication of this manuscript now and in the future. We don’t hold and currently applying for any patents relating to the content of the manuscript. We didn’t receive reimbursements, fees, funding, or salary from an organization that holds or has applied for patents relating to the content of the manuscript. We don’t have any other financial competing interests. The authors declare that they have no non-financial competing interests.

## Authors’ contributions

YDF performed the basic research, did statistical analysis and drafted this manuscript. WQD, LXB and LZJ made substantial contributions in data acquisition and data interpretation. WYX provided guidance throughout this process. As corresponding author, YSX designed and coordinated this research. All authors read and approved the final manuscript.

## Authors’ information

YSX: Professor & Director, Department of Radiation Oncology, the First Affiliated Hospital, College of Medicine, Zhejiang University, Zhejiang, China.

WYX: Associate Professor & Supervisor, Medical Imaging Laboratory, Department of Imaging and Interventional Radiology, The Chinese University of Hong Kong, Hong Kong, China.

## Pre-publication history

The pre-publication history for this paper can be accessed here:

http://www.biomedcentral.com/1471-2407/13/548/prepub
